# Metacognitive Short-Term Intervention in Patients With Mental Disorders Following Cardiovascular Events

**DOI:** 10.3389/fpsyt.2022.812807

**Published:** 2022-04-04

**Authors:** Philippa Gebhardt, Flora Caldarone, Mechthild Westhoff-Bleck, Karen M. Olsson, Marius M. Hoeper, Da-Hee Park, Britta Stapel, Michael H. Breitner, Oliver Werth, Ivo Heitland, Kai G. Kahl

**Affiliations:** ^1^Department of Psychiatry, Social Psychiatry and Psychotherapy, Hannover Medical School, Hannover, Germany; ^2^Department of Cardiology and Angiology, Hannover Medical School, Hannover, Germany; ^3^Department of Respiratory Medicine, Hannover Medical School, Member of the German Center for Lung Research (DZL/BREATH), Hannover, Germany; ^4^Biomedical Research in Endstage and Obstructive Lung Disease Hannover (BREATH), Hannover, Germany; ^5^Information Systems Institute, Leibniz University Hannover, Hannover, Germany

**Keywords:** cardiovascular disease, mental disorder, metacognitive therapy, psychotherapy, psychocardiology

## Abstract

**Background:**

Mental disorders are common among patients with severe cardiovascular diseases (CVD). Yet, there is a lack of easily accessible evidence-based treatments. Recent research indicates elevated prevalence of dysfunctional metacognitions in patients with mental disorders following cardiovascular events. As metacognitive therapy (MCT) is an established treatment to modify metacognitions, we tested if a brief metacognitive intervention *via* videotelephony is effective in this patient group.

**Methods:**

A brief MCT treatment was tailored to CVD patients and designed as a face-to-face internet-based intervention. Five patients with CVDs and comorbid mental disorders underwent a psychocardiological examination and diagnostic approach. Each patient participated in eight 50 min sessions *via* encrypted video messenger service. Metacognitions, depression and anxiety symptoms and quality of life were assessed by self-report measures pre- and post-treatment. Patients rated dysfunctional thought processes, current psychological impairment, and treatment satisfaction after each session. Intended follow-up measures were not reported due to missing data.

**Results:**

For most patients, the brief metacognitive intervention was associated with a decrease in dysfunctional metacognitions and a reduction of symptoms of anxiety and depression post-treatment. Psychological and physiological quality of life improved. Patients reported high satisfaction with the tailored treatment.

**Conclusion:**

Our results suggest that a brief internet-based metacognitive treatment may be a promising tool for patients with CVDs and comorbid mental disorders. Feasibility and acceptance of the intervention was rated high by the patients. Further research is necessary to support the preliminary findings and to adapt and evaluate the intervention in a controlled clinical trial setting.

## Introduction

The interaction of psychological and cardiovascular health is a subject of interest in many different fields of research. Studies regarding the comorbidity of cardiovascular and psychiatric diseases have shown that the prevalence of mental illnesses is increased across patients with different cardiovascular diseases (CVDs) (1–3). The most common disorders among CVD patients are pathological stress reactions after a cardiovascular event, depressive and anxiety disorders (3–6). Psychiatric symptoms and illnesses are in turn associated with several worse outcomes regarding the CVD: poorer prognosis of the course of the CVD, lower quality of life (QoL), increased risk of mortality, and interference with the CVD treatment, for example, due to worse adherence (3, 7–11). Many current guidelines for the different CVDs therefore include recommendations that vary from psychosocial support to psychotherapy, depending on the severity of the psychological distress (12–14). Offered psychological treatments, for example, for the most common CVD coronary heart disease (CHD), include a variation of components (e.g., relaxation techniques, cognitive strategies, psychological support) (15). Systematic reviews and meta-analyses report small to moderate effects of these psychological treatments on depressive and anxiety symptoms, but the quality of evidence was low to very low (15). Overall mortality and the risk of cardiovascular events were not affected by the treatments investigated (15). Richards et al. (16) also found no evidence that the currently used interventions of cognitive behavioral therapy in CHD patients, which is the gold standard in the treatment of anxiety and depression in general, were more effective than the other ones investigated. In clinical practice, only a fraction of CVD patients with a comorbid mental illness actually receives an adequate psychological treatment (5, 17, 18). Reasons for mental disorder treatment gaps in general include long waiting times and limited regional access (19, 20) which can plausibly be applied to mental disorders in the context of CVDs. Thus, there is the need for evidence-based, effective, and accessible treatments for CVD patients with mental disorders.

The metacognitive approach (21) provides a framework for mental disorders in CVD patients. Metacognitive theory (21, 22) posits that the cognitive processes that are involved in interpreting, monitoring, and controlling cognition (the so-called metacognitions) are transdiagnostically associated with mental disorders (23). A key role in the pathogenesis and maintenance of mental disorders is attributed to the Cognitive Attentional Syndrome (CAS): The CAS describes a dysfunctional cognitive pattern, characterized by inflexible attention, perseverative thinking like worry and rumination, threat monitoring and dysfunctional self-regulatory behaviors, e.g., avoidance (21). It is postulated to lead to the inability to process normal emotional distress and to result in its maintenance. One’s positive and negative metacognitive beliefs, which state the assumed usefulness and uncontrollability as well as danger of thoughts and processes involved in the CAS, respectively, lead to its progression (21, 23). Recent research exploring metacognitions in CVD patients suggests that the application of a metacognitive framework onto the psychological processes following a CVD poses a good fit, with repetitive negative thinking and metacognitive beliefs being found amongst patients with resulting mental disorders (24)^1^. In metacognitive therapy (MCT) (23), these maladaptive metacognitive processes and beliefs are modified to establish more flexible thinking and therefore reduce psychological distress. MCT is an effective treatment, especially for anxiety and depression (25). Recently, the effectiveness of MCT for mental disorders among somatically ill patients has been explored. Findings show that MCT is a feasible treatment with promising effects in that patient group as well (26, 27). There is first evidence from a single case study that a brief MCT in an individual setting is a beneficial treatment for a mental disorder in a patient with a CVD (28): The treatment consisted of several MCT techniques with a focus on attention training (29) and detached mindfulness (23). After four sessions, the patient with an adjustment disorder showed a full remission from it and improvements in MCT specific scores.

The aim of this study was to further explore the effects of an individual metacognitive therapy in patients with the most common mental disorders following different CVDs. Next to the advantages of the metacognitive approach, which allows for a transdiagnostic treatment in a short period of time, the treatment was internet-based as it was conducted *via* videotelephony. During the last years, a wider use of internet-based treatments has been established. They have several advantages: Studies have shown internet-based CBT-treatments to be effective for several mental disorders, amongst them anxiety and depressive disorders (30). They ease the problem of regional restrictions of availability of psychological interventions and might be a promising way of alleviating the accessibility problem. Furthermore, they are feasible and gained in importance during the SARS-CoV-2 pandemic (31, 32). Thus, the delivery of MCT *via* internet should be investigated as well to take advantage of the benefits (33). Taken together, this study aims to explore the effectiveness of a short-term metacognitive intervention *via* videotelephony in a sample of CVD patients.

## Methods

### Participants

Patients were referred by the Department of Cardiology and Angiology, the Department of Respiratory Medicine and the Department of Psychiatry, Social Psychiatry and Psychotherapy of Hannover Medical School. Patients were included if they met the following criteria: (1) confirmed diagnosis of a CVD, (2) minimum age of 18 years, (3) free of psychiatric medication or stable on medication for at least 3 months, (4) diagnosis of adjustment disorder, major depressive disorder and/or anxiety disorder according to DSM-V criteria, and (5) no acute suicidal tendency. All patients reported to fulfill the given criteria and provided written consent to be part of the study. For an overview of the somatic and psychiatric diagnoses of each patient, see Supplementary Table 1.

Initially, six patients were included. One patient received the treatment but was removed from the case series due to concurrent outpatient psychotherapy.

#### Patient 1

Patient 1 was a 40-year-old female who had been diagnosed with idiopathic pulmonary arterial hypertension (IPAH) three years prior. Additionally, she suffered from obesity, obstructive sleeping disorder, and endometriosis. She was diagnosed with major depressive disorder and a binge eating disorder in 2002. She received an 8-week cognitive behavioral therapy (CBT) at a day-clinic in 2004. Yet, since the IPAH diagnosis, depressive symptoms reoccurred. During inclusion, she met the diagnostic criteria for a depressive episode and persistent symptoms of the binge eating disorder. Patient 1 took anti-depressant medication (Venlafaxine) and did an outpatient CBT one year prior to the study. She had been unemployed for three years.

#### Patient 2

Patient 2 was a 38-year-old male who developed a two-vessel disease and suffered a myocardial infarction three and a half years prior to inclusion in this study. He received a stent for one vessel plus an implantable cardioverter-defibrillator as a treatment. The patient received a 7-week inpatient psychotherapy followed by two and a half years of outpatient psychotherapy after the cardiac event due to depression (still present during this study), panic disorder and reactivation of traumatic events from his youth. During inclusion, he was employed. He was free of medication and received no psychotherapy.

#### Patient 3

Patient 3 was a 54-year-old female who was diagnosed with IPAH in 2019. In addition, she suffered from fibromyalgia, diabetes mellitus type 2, rheumatic arthritis, polyneuropathy, gout, migraines, and obesity. The patient received a 3-month inpatient treatment due to depression followed by two years of outpatient psychotherapy before the IPAH diagnosis. She received a disability pension due to her somatic illnesses since 2019. During inclusion, she suffered from a depressive episode and was free of psychopharmacological medication.

#### Patient 4

Patient 4 was a 42-year-old male who developed ventricular extrasystoles half a year prior to inclusion during a foreign assignment. He met the diagnostic criteria for an adjustment disorder and reported symptoms of anxiety and low mood. Patient 4 was not on psychiatric medication and has not received any kind of psychotherapy. He was still employed but was currently unable to continue his work abroad due to the psychological impairment. He had been on sick leave for one month during inclusion.

#### Patient 5

Patient 5 was a 65-year-old female with a 14-year history of chronic thromboembolic pulmonary hypertension. Since the diagnosis, she reported to suffer from severe health-related restrictions, e.g., limitations of movement and a low activity rate. During inclusion, she met the diagnostic criteria for a major depressive disorder and a generalized anxiety disorder. She was free of psychiatric medication; however, she previously received a 4-week inpatient CBT three years ago. Patient 5 was retired.

### Measures

#### Hospital Anxiety and Depression Scale

The Hospital Anxiety and Depression Scale (HADS) (34) is a self-report measure assessing anxiety and depressive symptoms over the last week. It consists of 14 items, which are rated on a 4-point Likert scale from 0 to 3. The questionnaire includes two subscales with seven items each, resulting in subscale-scores ranging from 0 to 21. Higher scores indicate higher levels of anxiety and depressive symptoms (34). The HADS has good psychometric properties and is commonly used, especially in patients with somatic illnesses (35, 36). For the study, the German version was used (37).

#### Metacognitions Questionnaire-30

The Metacognitions Questionnaire-30 (MCQ-30) (38) is a self-report scale that measures beliefs about worry, monitoring tendencies and thinking itself. It consists of five subscales: positive beliefs about worry, negative beliefs about uncontrollability of thoughts and danger, beliefs about cognitive competence, beliefs about need to control thoughts, and cognitive self-consciousness. 30 items are answered on a 4-point Likert scale from 1 (“do not agree”) to 4 (“agree very much”), giving a range from 30 to 120. Higher scores indicate a greater level of dysfunctional metacognitions. The instrument has good internal consistency, convergent, and construct validity and acceptable to good test-retest reliability (38). For the study, the German version of the MCQ-30 was used (39).

#### World Health Organization Quality of Life Assessment

The World Health Organization Quality of Life Assessment (WHOQOL-BREF) (40) is a short version of the WHOQOL-100 (41), and is a cross-cultural measurement of wellbeing and QoL. The assessment comprises four domains of QoL: physical health, psychological, social, and environment (40). In addition, one facet covering overall QoL and general health is assessed. The 26 items were self-rated referring to the last 2 weeks on a 5-point Likert scale. The sum scores of the domains are transformed on a scale from 0 to 100 with higher scores indicating a better QoL. The WHOQOL-BREF domain scores have demonstrated good discriminant validity, content validity, internal consistency, and test-retest reliability (40). For the study, the German version was used (42).

#### Visual Analog Scales

Visual analog scales (VAS) were used to evaluate the course of symptoms (e.g., self-focused attention, worry and rumination and perceived uncontrollability of thoughts) and treatment satisfaction. Patients rated nine items on a scale from 1 to 10. The VAS items are included in Supplementary Table 2.

### Procedure

Patients were contacted by one of three therapists to confirm inclusion and exclusion criteria. The initial session was used for a psychocardiological examination. Trained psychologists conducted diagnostic interviews to assess mental disorders according to DSM-V criteria. Subsequently, participants received seven sessions of 50 min duration twice a week. This was followed by two 25 min follow-up booster sessions after 6 and 12 weeks. All sessions were conducted online using an encrypted medical video messenger service. HADS, MCQ-30, and WHOQOL-BREF were completed at baseline, post-treatment and at 6 and 12-week follow-up. Additionally, the VAS were filled out at each treatment session.

### Treatment

The treatment protocol was based on the MCT manual by Wells (23). The treatment plan was tailored to the specific needs of a person suffering from the co-occurrence of somatic and mental illness. To that end, statements of cardiologists, the patients with CVDs and their relatives were collected and analyzed. Accordingly, a modular treatment plan was developed. The treatments were provided by three of the authors (FC, PG, IH) after receiving training and under continuous supervision for FC and PG. The treatment was planned as a short-term intervention which aimed to be economic but efficient. Therefore, we decided to heighten the frequency to twice weekly sessions as there is evidence for improved treatment outcomes (43). A frequent examination of the correct execution and adherence to the treatment exercises (e.g., ATT) was an additional reason for the twice weekly sessions.

The treatment plan included the following modules:

(1) Case formulation and Socialization
(2) Attention Training Technique (ATT)
(3) Detached Mindfulness (DM) strategies
(4) Challenging positive and negative metacognitive beliefs
(5) Modifying dysfunctional coping mechanisms
(6) Relapse prevention

**FIGURE 1:**
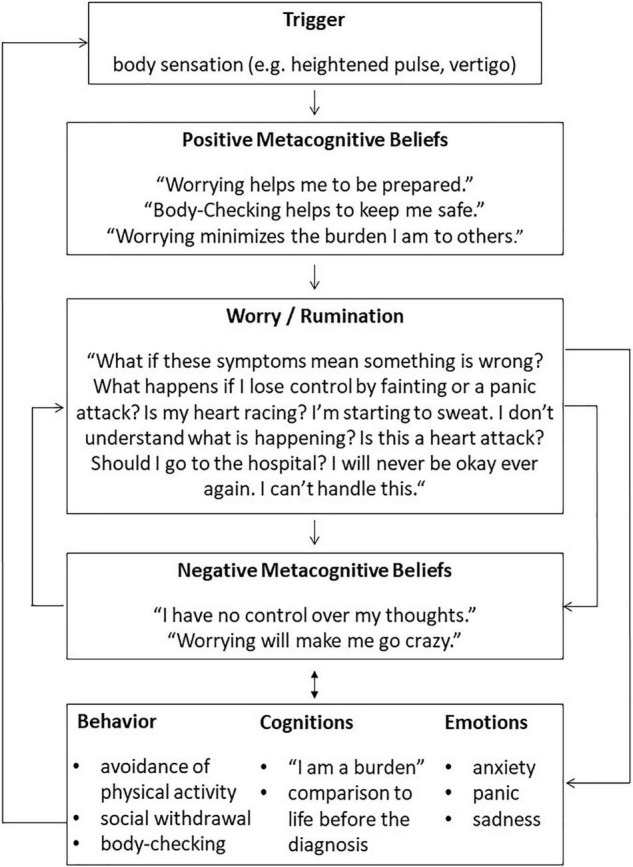
Exemplary case formulation for a patient with idiopathic pulmonary arterial hypertension based on Wells (23).

First, a personalized case conceptualization was drawn up using a transdiagnostic model adapted to the cardiovascular patients. Cardiovascular symptoms were incorporated with consequent thinking processes of the Cognitive Attentional Syndrome (CAS), such as worrying and an ongoing shift of attention to body sensations. Underlying positive and negative metacognitive beliefs were identified and linked to the reported thinking patterns. Consequences on an emotional, behavioral and cognitive level were discussed. For an exemplary case formulation, see Figure 1. The socialization to the model was supported by questions demonstrating the role of the different model elements and their circularity. The next step was the introduction of the ATT (29). Goal of the ATT is to strengthen attentional control, flexibility, and resources (23, 29). It has been shown to have the above mentioned attentional effects in anxiety, depression (44) as well as adjustment disorder in a CVD patient (28). Thus, the German Version of ATT was included in the treatment plan (23). Participants were asked to practice the ATT twice a day during the treatment. Next, DM was introduced using metaphors from the MCT manual (e.g., cloud or train metaphor) (23). DM focuses on the reactions to one’s own thinking processes (21). It aims to raise metacognitive awareness and a detached perspective to dysfunctional thoughts by not reacting to them on purpose (23). DM was applied using exercises (e.g., tiger task, prescriptive mind-wandering) during the sessions which were practiced independently throughout the intervention. Worry postponement was introduced and practiced by the participants in between the sessions. Further, positive and negative metacognitive beliefs were challenged: beliefs about the uncontrollability of thoughts were discussed in the context of the patients’ experiences with worry postponement. Given the severe cardiovascular illnesses of the patients, the distinction between unhelpful attentional responses to body sensations and necessary reactions in case of aggravating symptoms was discussed. Metacognitive socratic dialog was used to challenge beliefs about strategies like constant threat monitoring, e.g., checking for signs of vertigo and irregular heartbeats. Behavioral experiments were used to test their helpfulness as well as the short and long term consequences. The usefulness of remaining maladaptive coping mechanisms such as avoidance of physical activity like stair climbing and withdrawal were discussed. The “old plan–new plan” protocol (23) was used to build and strengthen new strategies. A list of potential triggers of the CAS was composed. Lastly, relapse prevention was provided by summarizing the main strategies of the treatment. The use of the new strategies in potentially challenging situations was discussed. During the treatment, the adherence to work between sessions (e.g., ATT, DM exercises) was verbally queried in the following session. In the follow-up sessions, the implementation of the learned strategies was discussed. Patients reported examples of the “new plan” protocol and occurring difficulties and questions were examined. The objective of the follow-up sessions was to consolidate the knowledge and strategies patients acquired during the treatment. Due to the short intervention, the follow-ups were planned as short booster sessions shortly after the completion of the eight treatment sessions.

### Data Analysis

To investigate whether the intervention affected metacognitions and psychological symptoms, visual analyses were used. Graphical representation is a common way to present data in case-series-research. Parsonson and Baer (45) argue that using visual analysis of graphs, only clear and distinguishable effects will be noted. Therefore, bar graphs are used to present the scores of the questionnaires (HADS, MCQ-30, WHOQOL-BREF) pre- and post-treatment for each patient. Additionally, the percentage of improvement was calculated for each patient. As the WHOQOL-BREF score is transformed into percentages, the change of that score is presented. Patient 2 did not attend the follow-up sessions, patients 1, 2, and 3 did not fully complete the follow-up questionnaires. Due to the amount of missing data and potential selection effects, the follow-up data is not reported. The courses over each session assessed by VAS are illustrated as line graphs for each patient. For patient 2, VAS values are missing at session 3.

To determine whether the change that occurred during the treatment was clinically significant, we used a two-step criterion proposed by Jacobson and Truax (46): First, it is evaluated if the patients are part of the functional or dysfunctional group after the treatment, based on their provided questionnaires scores. Hermann-Lingen, Buss, and Snaith (37) reported standard cut-off scores of <8 for both HADS-subscales, which were shown to have good sensitivity and specificity for identifying possible anxiety or depressive disorders (37). In the second step, the reliable change index (RCI) is used to determine whether the amount of change that occurred was reliable. When the calculated RC score is >1.96, it is unlikely that the change that occurred during the treatment does not reflect real change (46). The data needed for its calculation was drawn from the German HADS manual as well (37). Based on the two-step criterion, patients’ changes are then classified as recovered, if they pass both the cut-off and the RC criterion, as improved, if they only pass the RC criterion, as unchanged, if they pass neither, or as deteriorated, if their scores worsened and passed the RC criterion (46).

## Results

**FIGURE 2:**
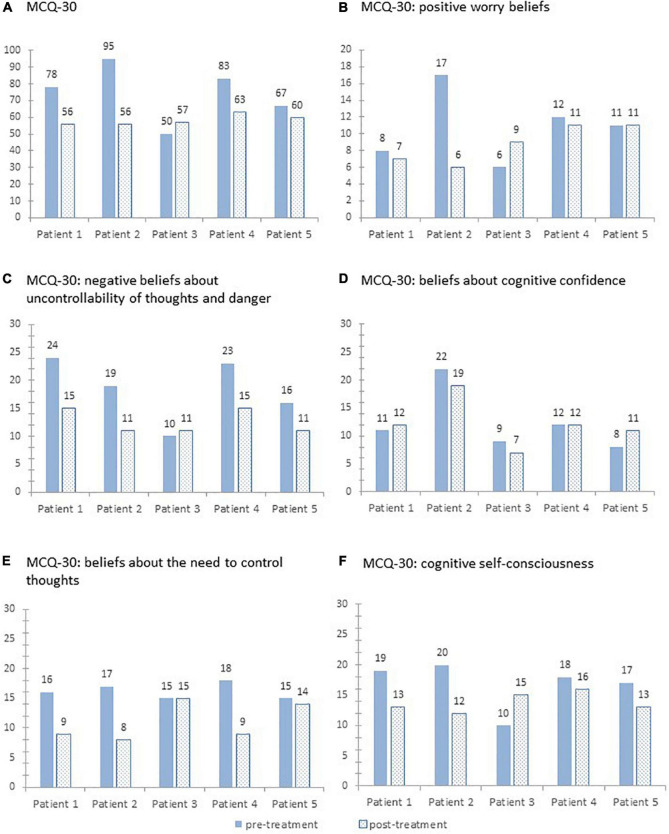
Sum scores on the Metacognitions Questionnaire-30 **(A)** and its five subscales **(B–F)** pre- and post-treatment for each patient.

For the sum score of the MCQ-30, all patients except patient 3 showed a reduction from pre- to post-treatment. The percentage change ranged from +14 to −41%. The subscales of the MCQ-30 show a heterogeneous pattern for the individual patients. For details, see Figure 2.

**FIGURE 3:**
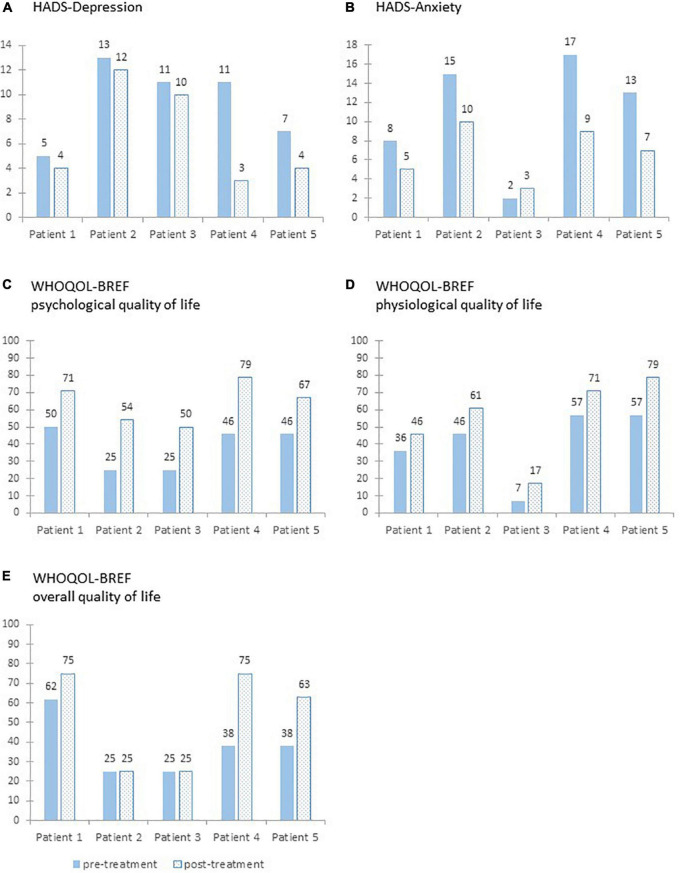
Sum scores on the two subscales of the Hospital Anxiety and Depression Scale **(A,B)** and transformed sum scores on the World Health Organization Quality of Life Assessment **(C–E)** pre- and post-treatment for each patient.

For the HADS-D, all patients showed a reduction from pre- to post-treatment (see Figure 3). The percentage change ranged from −8 to −72%. For HADS-A, there was a reduction from pre- to post-treatment for four patients. One patient reported increasing scores. The percentage change ranged from +50 to −47%.

Regarding the clinical significance of the changes, only patient 4 can be classified as recovered for the HADS-D. All other patients did not meet the RC criterion and can therefore be classified as unchanged. However, HADS-D scores of patient 1 and 5 were in the functional group pre- and post-treatment. In contrast, patient 5 can be classified as recovered and patient 2 and 4 can be classified as improved concerning the anxiety scores. Patient 1 and 3 were classified as unchanged; however, their HADS-A post-treatment scores were below the given cut-off.

For the WHOQOL-BREF (see Figure 3), the scores of the subscale psychological QoL increased from pre- to post-treatment for all patients. The improvement in the psychological QoL ranged from +21 to +33. The scores of the subscale physiological QoL increased for all patients. The improvement in the physiological QoL ranged from +10 to +22. For the overall QoL, the pre- to post-treatment scores remained stable for two patients and increased for the other three patients. The improvement in the overall QoL from ranged from ±0 to +37.

**FIGURE 4:**
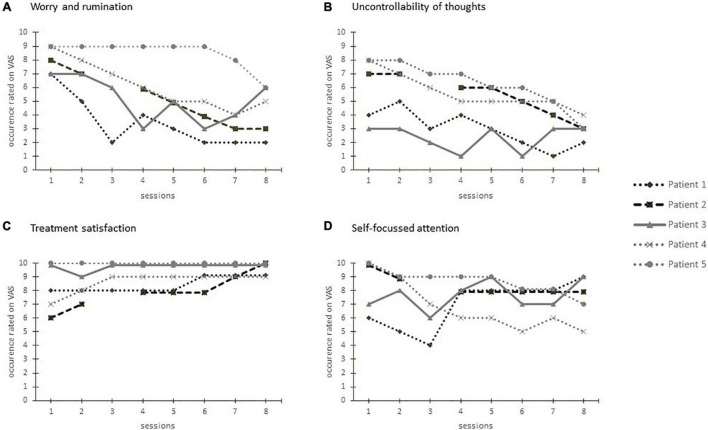
Visual analog scales regarding worry and rumination **(A)**, perceived uncontrollability of thoughts **(B)**, treatment satisfaction **(C)**, and self-focused attention **(D)** after each treatment session for each patient.

The courses of the VAS during the sessions for each patient are shown in Figure 4. Patients reported to be highly affected by worry and rumination at the beginning of the treatment. Over the course of the intervention, patient 3 reported fluctuating levels of worry and rumination. The scores of patient 1, 2, 4, and 5 showed a tendency toward reduction. At the beginning of the treatment the perceived uncontrollability of thoughts varied widely. Patient 1 and 3 who reported a comparatively low level of perceived uncontrollability showed a fluctuating course on a consistent level. Patient 1, 2, and 4 reported a higher level of uncontrollability at first and showed a continuous reduction over the course of the intervention. The course of the reported self-focused attention is heterogeneous with continuously high values for patient 1 and 2, decreasing scores for patient 4 and an increasing course for patients 3 and 5. The reported treatment satisfaction was overall high for patients 1 and 3, the three other patients reported an increasing satisfaction over the course of the treatment.

## Discussion

We developed a brief metacognitive treatment adapted to the needs of CVD patients with consecutive mental disorders. Further, we investigated the effects of the treatment delivered face-to-face *via* videotelephony.

After the treatment, most patients reported a reduction of dysfunctional metacognitions, improved psychological QoL and a reduction of anxiety and depressive symptoms. According to the criteria regarding clinical significance of change, particularly anxiety symptoms were improved after the psychotherapeutic intervention, with three out of five patients displaying recovery or improvement. Our data suggests that a tailored metacognitive approach could be suitable for CVD patients, as it seemed to target the intended mechanisms. This is consistent with studies showing the effectiveness of MCT for patients with somatic diseases that caused or worsened psychiatric disorders (26–28). As this case series only included five patients, the tailored treatment should be further investigated in a randomized controlled setting to probe the preliminary effects. Despite the promising tendency, there is room for improvement regarding the amount of reduced psychological symptoms and dysfunctional metacognitions. A possible reason for the effects observed in our patient sample may be the standardized procedure in a clinically heterogeneous group of patients. A treatment adaptation to the specific psychiatric symptoms consecutive to the underlying CVD could be used in further research.

The findings indicate that there might be some characteristic dysfunctional metacognitions that are associated with the specific somatic symptoms of CVDs. For example, as shown in the model (see Figure 1), for anxiety and depressive symptoms developed after an IPAH diagnosis, there are triggers induced or related to the CVD. An exemplary trigger is an increased pulse rate. The association of metacognitions and the underlying somatic illnesses supports the findings of prominent health worry among certain CVD patients (see text footnote 1). During the individual case conceptualizations, different foci became apparent: For some patients, self-focused attention and body-checking were most present, others reported to suffer most from rumination. This was also reflected in the heterogeneous courses of the MCQ-30 subscales. Therefore, the modular structure of the treatment plan could be used to prioritize the most prominent dysfunctional metacognitions and maladaptive coping behaviors. This is in agreement with Wells (23), who suggests using adapted versions of the generic MCT model and treatment, depending on the diagnoses.

The treatment was based on eight twice-weekly sessions. The given reductions of psychological symptoms and improvement of QoL in the short amount of time indicate the usefulness of brief treatments presented in a high frequency. This opens up potential for short interventions to efficiently improve psychological health, for example, shortly after a diagnosis of a CVD.

We tested the brief CVD-tailored metacognitive treatment *via* videotelephony. Treatment satisfaction was overall high and no apparent problems were reported or occurred. Thus, an internet-based approach seems to be a feasible way to apply MCT. Especially considering the physical constraint accompanying CVDs and the additional restrictions during the SARS-CoV-2 pandemic, internet-based treatments like the present one are a necessary mean to enhance traditional face-to-face psychotherapy.

Our study has limitations: The small sample size and absence of a control group limits the generalizability of the findings. The concurrent pharmacological treatments may distort the results. The lack of a no-treatment baseline limits the interpretability of the treatment effects. Due to the missing data for the follow-up period, the long-term stability of the effects is unclear. The treatment was based on the manual by Wells (23) and the execution was supervised, but there was no rating of therapist adherence. Patients verbally reported a high adherence to the treatment exercises but there was no standardized measurement to control the proper execution.

In conclusion, the outcomes provide preliminary evidence that an internet-based metacognitive approach might be promising for treating psychological distress following CVDs. The treatment plan, including the duration and adaptation to psychiatric as well as cardiovascular diagnoses, should be further investigated by using randomized and controlled studies.

## Data Availability Statement

The raw data supporting the conclusions of this article will be made available by the authors, without undue reservation.

## Ethics Statement

The studies involving human participants were reviewed and approved by Hannover Medical School Ethics Committee. The patients/participants provided their written informed consent to participate in this study.

## Author Contributions

FC and PG were responsible for study design, implementation of the study, data collection, statistical analysis, data interpretation, and drafted the manuscript. MW-B, D-HP, KO, and MH were responsible for implementation of the study and data collection. IH was responsible for study design, implementation of the study, data collection, statistical analysis, data interpretation, and revised the manuscript. KK was responsible for study design, implementation of the study, data interpretation, and revised the manuscript. All authors contributed to the article and approved the submitted version.

## Conflict of Interest

KO has received fees for lectures and/or consultations from Actelion, Janssen, MSD, Bayer, United Therapeutics, GSK, Janssen, Pfizer, and Acceleron, all outside the present study. MH has received honoraria for lectures and/or consultations from Acceleron, Actelion, Bayer, GSK, Janssen, MSD, and Pfizer, all outside the present study. D-HP has received honoraria for lectures from Janssen. KK has received honoraria for consultations and/or lectures from Eli Lilly, Janssen, Lundbeck, Neuraxpharm, Otsuka, Pfizer, Servier, Schwabe, Takeda, and Trommsdorff/Ferrer, Alexion, and CannaXan (advisory board). The remaining authors declare that the research was conducted in the absence of any commercial or financial relationships that could be construed as a potential conflict of interest.

## Publisher’s Note

All claims expressed in this article are solely those of the authors and do not necessarily represent those of their affiliated organizations, or those of the publisher, the editors and the reviewers. Any product that may be evaluated in this article, or claim that may be made by its manufacturer, is not guaranteed or endorsed by the publisher.

## Footnotes

1 Caldarone F, Gebhardt P, Hoeper MM, Olsson KM, Fuge J, Park D-H, et al. Metacognitions in patients with frequent mental disorders after diagnosis of pulmonary arterial hypertension. *Front Psychiatry*. (under review).

## References

[1] Thombs BD Bass EB Ford DE Stewart KJ Tsilidis KK Patel U Prevalence of depression in survivors of acute myocardial infarction: review of the evidence. *J Gen Intern Med.* (2006) 21:30–8. 10.1111/j.1525-1497.2005.00269.x 16423120 PMC1484630

[2] Patel D Mc Conkey ND Sohaney R Mc Neil A Jedrzejczyk A Armaganijan LA. Systematic review of depression and anxiety in patients with atrial fibrillation: the mind-heart link. *Cardiovasc Psychiatry Neurol.* (2013) 2013:1–11. 10.1155/2013/159850 23710335 PMC3655604

[3] Olsson KM Meltendorf T Fuge J Kamp JC Park D-H Richter MJ Prevalence of mental disorders and impact on quality of life in patients with pulmonary arterial hypertension. *Front Psychiatry.* (2021) 12:667602. 10.3389/fpsyt.2021.667602 34135787 PMC8200462

[4] Edmondson D Richardson S Falzon L Davidson KW Mills MA Neria Y. Posttraumatic stress disorder prevalence and risk of recurrence in acute coronary syndrome patients: a meta-analytic review. *PLoS One.* (2012) 7:e38915. 10.1371/journal.pone.0038915 22745687 PMC3380054

[5] Huffman JC Celano CM Beach SR Motiwala SR Januzzi JL. Depression and cardiac disease: epidemiology, mechanisms, and diagnosis. *Cardiovasc Psychiatry Neurol.* (2013) 2013:1–14. 10.1155/2013/695925 23653854 PMC3638710

[6] Tully PJ Cosh SM. Generalized anxiety disorder prevalence and comorbidity with depression in coronary heart disease: a meta-analysis. *J Health Psychol.* (2013) 18:1601–16. 10.1177/1359105312467390 23300050

[7] Roest AM Martens EJ Denollet J de Jonge P. Prognostic association of anxiety post myocardial infarction with mortality and new cardiac events: a meta-analysis. *Psychosom Med.* (2010) 72:563–9. 10.1097/PSY.0b013e3181dbff97 20410247

[8] Gathright EC Goldstein CM Josephson RA Hughes JW. Depression increases the risk of mortality in patients with heart failure: a meta-analysis. *J Psychosom Res.* (2017) 94:82–9. 10.1016/j.jpsychores.2017.01.010 28183407 PMC5370194

[9] Tully PJ Bennetts JS Baker RA McGavigan AD Turnbull DA Winefield HR. Anxiety, depression, and stress as risk factors for atrial fibrillation after cardiac surgery. *Heart Lung.* (2011) 40:4–11. 10.1016/j.hrtlng.2009.12.010 20561864

[10] Zhang WY Nan N Song XT Tian JF Yang XY. Impact of depression on clinical outcomes following percutaneous coronary intervention: a systematic review and meta-analysis. *BMJ Open.* (2019) 9:e026445. 10.1136/bmjopen-2018-026445 31434764 PMC6707663

[11] Halling T Akkermann S Löffler F Groh A Heitland I Haefeli WE Factors that influence adherence to medication in adults with congenital heart disease (ACHD). *Front Psychiatry.* (2021) 12:788013. 10.3389/fpsyt.2021.788013 34899440 PMC8660073

[12] Visseren FLJ Mach F Smulders YM Carballo D Koskinas KC Bäck M 2021 ESC guidelines on cardiovascular disease prevention in clinical practice. *Eur Heart J.* (2021) 42:3227–37. 34458905 10.1093/eurheartj/ehab484

[13] McDonagh TA Metra M Adamo M Gardner RS Baumbach A Böhm M 2021 ESC guidelines for the diagnosis and treatment of acute and chronic heart failure. *Eur Heart J.* (2021) 42:3599–726. 34447992 10.1093/eurheartj/ehab368

[14] Galiè N Humbert M Vachiery J-L Gibbs S Lang I Torbicki A 2015 ESC/ERS guidelines for the diagnosis and treatment of pulmonary hypertension: the joint task force for the diagnosis and treatment of pulmonary hypertension of the European society of cardiology (ESC) and the European respiratory society (ERS)Endorsed by: association for European paediatric and congenital cardiology (AEPC), international society for heart and lung transplantation (ISHLT). *Eur Heart J.* (2016) 37:67–119. 10.1093/eurheartj/ehv317 26320113

[15] Richards SH Anderson L Jenkinson CE Whalley B Rees K Davies P Psychological interventions for coronary heart disease: cochrane systematic review and meta-analysis. *Eur J Prev Cardiol.* (2018) 25:247–59. 10.1177/2047487317739978 29212370

[16] Richards SH Anderson L Jenkinson CE Whalley B Rees K Davies P Psychological interventions for coronary heart disease. *Cochrane Database Syst Rev.* (2017) 4:CD002902. 10.1002/14651858.CD002902.pub4 28452408 PMC6478177

[17] Celano CM Villegas AC Albanese AM Gaggin HK Huffman JC. Depression and anxiety in heart failure: a review. *Harv Rev Psychiatry.* (2018) 26:175–84. 10.1097/HRP.0000000000000162 29975336 PMC6042975

[18] Löwe B Gräfe K Ufer C Kroenke K Grünig E Herzog W Anxiety and depression in patients with pulmonary hypertension. *Psychosom Med.* (2004) 66:831–6. 10.1097/01.psy.0000145593.37594.39 15564346

[19] Bundespsychotherapeutenkammer. *Wartezeiten 2018 [Internet].* (2018). Available online at: https://www.bptk.de/wp-content/uploads/2019/01/20180411_BPtK-Studie_Wartezeiten_2018.pdf (accessed October 20, 2021).

[20] Robert Koch-Institut. *Inanspruchnahme Psychiatrischer und Psychotherapeutischer Leistungen – Individuelle Determinanten und Regionale Unterschiede.* (2019). Available online at: https://edoc.rki.de/handle/176904/2899.2 (accessed October 27, 2021).

[21] Wells A Matthews G. *Attention and Emotion: A Clinical Perspective.* Hillsdale, NJ: Lawrence Erlbaum Associates, Inc (1994).

[22] Wells A Matthews G. Modelling cognition in emotional disorder: the S-REF model. *Behav Res Ther.* (1996) 34:881–8. 10.1016/s0005-7967(96)00050-2 8990539

[23] Wells A. *Metacognitive Therapy for Anxiety and Depression.* New York, NY: Guilford Press (2009).

[24] McPhillips R Salmon P Wells A Fisher P. Qualitative analysis of emotional distress in cardiac patients from the perspectives of cognitive behavioral and metacognitive theories: why might cognitive behavioral therapy have limited benefit, and might metacognitive therapy be more effective? *Front Psychol.* (2019) 9:2288. 10.3389/fpsyg.2018.02288 30662413 PMC6328488

[25] Normann N Morina N. The efficacy of metacognitive therapy: a systematic review and meta-analysis. *Front Psychol.* (2018) 9:2211. 10.3389/fpsyg.2018.02211 30487770 PMC6246690

[26] Wells A Reeves D Heal C Fisher P Davies L Heagerty A Establishing the feasibility of group metacognitive therapy for anxiety and depression in cardiac rehabilitation: a single-blind randomized pilot study. *Front Psychiatry.* (2020) 11:582. 10.3389/fpsyt.2020.00582 32714216 PMC7344162

[27] Fisher PL Byrne A Fairburn L Ullmer H Abbey G Salmon P. Brief metacognitive therapy for emotional distress in adult cancer survivors. *Front Psychol.* (2019) 10:162. 10.3389/fpsyg.2019.00162 30766505 PMC6365419

[28] Winter L Naumann F Olsson K Fuge J Hoeper MM Kahl KG. Metacognitive therapy for adjustment disorder in a patient with newly diagnosed pulmonary arterial hypertension: a case report. *Front Psychol.* (2020) 11:143. 10.3389/fpsyg.2020.00143 32116944 PMC7028769

[29] Wells A. Panic disorder in association with relaxation induced anxiety: An attentional training approach to treatment. *Behav Ther.* (1990) 21:273–80. 10.1016/j.janxdis.2008.10.008 19059753

[30] Carlbring P Andersson G Cuijpers P Riper H Hedman-Lagerlöf E. Internet-based vs. face-to-face cognitive behavior therapy for psychiatric and somatic disorders: an updated systematic review and meta-analysis. *Cogn Behav Ther.* (2018) 47:1–18. 10.1080/16506073.2017.1401115 29215315

[31] Tönnies J Hartmann M Wensing M Szecsenyi J Peters-Klimm F Brinster R Mental health specialist video consultations versus treatment-as-usual for patients with depression or anxiety disorders in primary care: randomized controlled feasibility trial. *JMIR Ment Health.* (2021) 8:e22569. 10.2196/22569 33709931 PMC7998325

[32] Beck-Hiestermann FML Kästner D Gumz A. Onlinepsychotherapie in zeiten der corona-pandemie. *Psychotherapeut.* (2021) 66:372–81. 10.1007/s00278-021-00519-0 34248286 PMC8256402

[33] McEvoy PM. Metacognitive therapy for anxiety disorders: a review of recent advances and future research directions. *Curr Psychiatry Rep.* (2019) 21:29. 10.1007/s11920-019-1014-3 30880368

[34] Zigmond AS Snaith RP. The hospital anxiety and depression scale. *Acta Psychiatr Scand.* (1983) 67:361–70. 10.1111/j.1600-0447.1983.tb09716.x 6880820

[35] Bjelland I Dahl AA Haug TT Neckelmann D. The validity of the hospital anxiety and depression scale. *J Psychosom Res.* (2002) 52:69–77. 10.1016/S0022-3999(01)00296-3 11832252

[36] Herrmann C. International experiences with the hospital anxiety and depression scale-a review of validation data and clinical results. *J Psychosom Res.* (1997) 42:17–41. 10.1016/s0022-3999(96)00216-4 9055211

[37] Hermann-Lingen C Buss U Snaith RP. *Hospital Anxiety and Depression Scale - Deutsche Version. Deutsche Adaptation der Hospital Anxiety and Depression Scale (HADS) von R. P. Snaith und A. S. Zigmond.* 3. ed. Bern: Hans Huber (2011).

[38] Wells A Cartwright-Hatton S. A short. *Behav Res Ther.* (2004) 42:385–96. 10.1016/S0005-7967(03)00147-5 14998733

[39] Wells A Schweiger U Wells A. *Metakognitive Therapie bei Angststörungen und Depression. 1. Aufl.* Basel: Beltz (2011).

[40] The WHOQOL Group. Development of the world health organization WHOQOL-BREF quality of life assessment. *Psychol Med.* (1998) 28:551-8. 10.1017/s0033291798006667 9626712

[41] The WHOQOL Group. The World Health Organization quality of life assessment (WHOQOL): development and general psychometric properties. *Soc Sci Med.* (1998) 46:1569–85. 10.1016/s0277-9536(98)00009-4 9672396

[42] Angermeyer M Kilian R Matschinger H. *WHOQOL - 100 und WHOQOL - BREF: Handbuch für die Deutschsprachige Version der WHO Instrumente zur Erfassung von Lebensqualität.* Göttingen: Hogrefe (2000).

[43] Bruijniks SJE Lemmens LHJM Hollon SD Peeters FPML Cuijpers P Arntz A The effects of once- versus twice-weekly sessions on psychotherapy outcomes in depressed patients. *Br J Psychiatry.* (2020) 216:222–30. 10.1192/bjp.2019.265 32029012

[44] Knowles MM Foden P El-Deredy W Wells AA. Systematic review of efficacy of the attention training technique in clinical and nonclinical samples: journal of clinical psychology. *J Clin Psychol.* (2016) 72:999–1025. 10.1002/jclp.22312 27129094

[45] Parsonson BS Baer DM. The visual analysis of data, and current research into the stimuli controlling it. In: Kratochwill TR Levin JR editors. *Single-Case Research Design and Analysis (Psychology Revivals).* London: Routledge (1992).

[46] Jacobson NS Truax P. Clinical significance: a statistical approach to defining meaningful change in psychotherapy research. *J Consult Clin Psychol.* (1991) 59:12–9. 10.1037/0022-006x.59.1.12 2002127

