# Supplementary material for: Metacognitive Short-Term Intervention in Patients With Mental Disorders Following Cardiovascular Events
Source: Front Psychiatry. 2022 Apr 4;13:812807. doi: 10.3389/fpsyt.2022.812807 (PMC9013742; doi:10.3389/fpsyt.2022.812807)
Supplement: Supplementary file 1 [file Data_Sheet_1.pdf]

Supplementary Material

Supplementary Table 1. Somatic and psychiatric diagnoses for all patients.

Patient	Somatic diagnoses	Psychiatric diagnoses
1	idiopathic pulmonary arterial hypertension, obesity, obstructive sleeping disorder, endometriosis	major depressive disorder, binge eating disorder
2	two-vessel disease, myocardial infarction	major depressive disorder, panic disorder
3	idiopathic pulmonary arterial hypertension, fibromyalgia, diabetes mellitus type 2, rheumatic arthritis, polyneuropathy, gout, migraines, obesity	major depressive disorder
4	ventricular extrasystoles	adjustment disorder
5	chronic thromboembolic pulmonary hypertension	major depressive disorder, generalized anxiety disorder

Supplementary Table 2. Description of the Visual analogue scales (VAS).

Topic	Question rated on VAS	VAS rating
Worry and rumination	How much are worry and rumination currently present?	from 1 (“not at all”) to 10 (“very much”)
Uncontrollability of thoughts	How do you currently perceive worry and rumination to be?	from 1 (“uncontrollable”) to 10 (“very controllable”); item was inverted
Self-focused attention	How strong do you perceive your self-focused attention on physical changes / complaints to be?	from 1 (“no self-focused attention”) to 10 (“very strong self-focused attention”)
Anxiety	How much are you currently affected by anxiety?	from 1 (“not at all”) to 10 (“very much”)
Anhedonia	How much are you currently affected by loss of interest and pleasure?	from 1 (“not at all”) to 10 (“very much”)
Physiological symptoms	How much are you currently affected by physiological complaints?	from 1 (“not at all”) to 10 (“very much”)
Treatment satisfaction	How satisfied are you with the brief psychotherapeutic treatment?	from 1 (“very unsatisfied”) to 10 (“very satisfied”)
Change of perspective	Did the so far treated subjects help you gain a new perspective on your symptoms?	from 1 (“not at all”) to 10 (“very much”)
Goal attainment	How much do the treated subjects help you to achieve your desired changes?	from 1 (“not at all”) to 10 (“very much”)

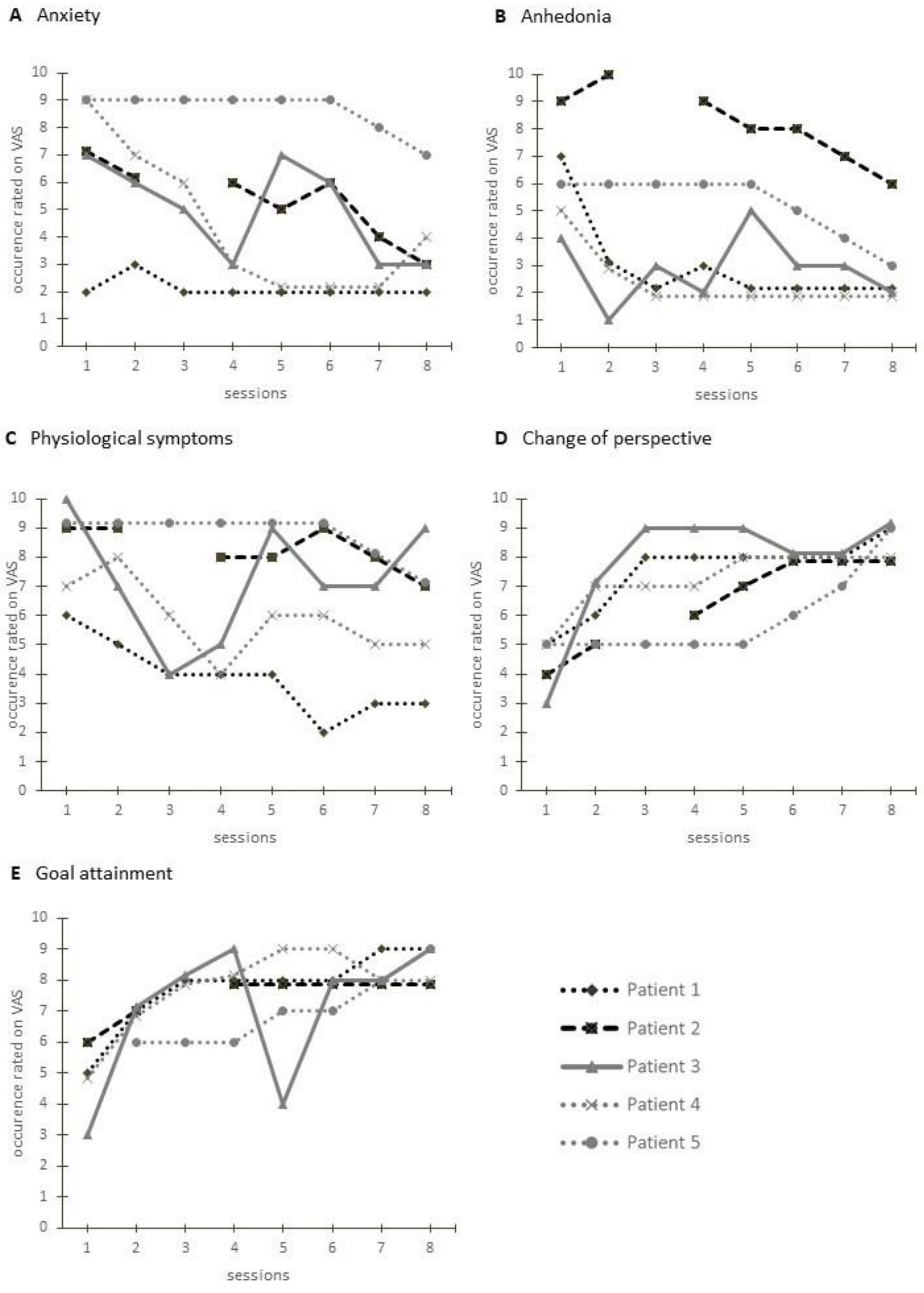

Supplementary Figure 1. Visual analog scales regarding symptoms of anxiety **(A)** and anhedonia **(B)**, physiological symptoms **(C)**, change of perspective **(D)** and goal attainment **(E)** after each treatment session for each patient.
